# Uncovering age-specific subtypes of pediatric obesity and metabolic syndrome using machine learning algorithms

**DOI:** 10.1038/s41598-025-24524-4

**Published:** 2025-11-19

**Authors:** Elahe Mousavi, Nafiseh Mozafarian, Motahar Heidari-Beni, Mohammadreza Sehhati, Roya Kelishadi

**Affiliations:** 1 Department of Bioinformatics, School of Advanced Technologies in Medicine, Isfahan University of Medical Sciences, Isfahan, Iran; 2 Child Growth and Development Research Center, Research Institute for Primordial Prevention of Non-Communicable Disease, Isfahan University of Medical Sciences Isfahan, Isfahan, Iran; 3 Department of Nutrition, Child Growth and Development Research Center, Research Institute for Primordial Prevention of Non-Communicable Disease, Isfahan University of Medical Sciences, Isfahan, Iran

**Keywords:** Obesity, Metabolic syndrome, Machine learning, Clustering, Computational biology and bioinformatics, Diseases, Endocrinology, Health care, Medical research, Risk factors

## Abstract

Identifying new subgroups among children and adolescents with obesity and metabolic syndrome requires advanced clustering techniques capable of analyzing complex multidimensional data. This study aimed to employ machine learning methods to enhance the classification of obesity and metabolic syndrome subgroups in youth, facilitating early detection and targeted intervention strategies. Data were derived from three nationwide, multicenter, school-based CASPIAN studies conducted in Iran during 2003–2004, 2009–2010, and 2015. After excluding metabolically healthy non-obese participants, the final sample included 382, 787, and 594 individuals aged 7–10, 11–14, and 15–18 years, respectively. Metabolic syndrome (MetS) status was defined according to Adult Treatment Panel III criteria. Unsupervised machine learning, specifically Gaussian Mixture Models (GMM), was applied to the top five principal components in each age group. The Davies-Bouldin index determined the optimal number of clusters. Clinical features associated with metabolism and obesity were analyzed within each cluster. In the 7–10 years group, six distinct clusters were identified based on key metabolic and anthropometric variables. The 11–14 years group yielded seven clusters, each with unique metabolic and anthropometric characteristics. For adolescents aged 15–18, six clusters reflected a more pronounced interaction between anthropometric measures and metabolic risk factors, consistent with physiological maturation. Stability tests showed mean clustering accuracies of 76.3%, 65.5%, and 52% for the three age groups, respectively. Predictability tests demonstrated an average accuracy exceeding 87% across all groups, indicating the robustness and reliability of the clustering approach. This study demonstrated that machine learning can uncover hidden metabolic and anthropometric heterogeneity in pediatric obesity, providing a methodological framework for identifying meaningful subgroups for targeted interventions.

**Supplementary Information:**

The online version contains supplementary material available at 10.1038/s41598-025-24524-4.

## Introduction

The increasing prevalence of obesity among children and adolescents has emerged as a major global public health concern. This trend is accompanied by a parallel rise in metabolic syndrome (MetS), a cluster of conditions that elevate the risk of cardiovascular disease (CVD) and type 2 diabetes mellitus (T2DM). The strong association between pediatric obesity and MetS underscores the importance of early identification and intervention strategies to reduce long-term health risks.

Recent studies indicate that the prevalence of MetS among obese children has reached concerning levels, with estimates ranging from 11.9% in overweight children to as high as 60% in those with obesity^1,2^. These findings highlight the substantially increased risk of serious health complications in children with MetS as they progress into adulthood^3^.

Traditional methods to defining obesity, particularly single-dimensional metrics such as body mass index (BMI), fail to capture the complexity of this multifaceted disease^4^. Although BMI remains a useful tool for initial screening, it does not accurately reflect the heterogeneous metabolic profiles and health statuses observed among individuals with obesity^5,6^. Moreover, the classification of individuals as metabolically healthy or unhealthy is highly variable, with reported prevalence rates of metabolic abnormalities among those classified as unhealthy ranging from 25% to 94%^7,8^.

These challenges underscore the need for more precise, data-driven approaches to identifying subgroups of obesity and metabolic risk, particularly in pediatric populations. In this context, machine learning (ML)—a subset of artificial intelligence (AI)—offers a powerful means of uncovering hidden patterns within complex, multidimensional datasets. ML algorithms can learn from data to detect underlying structures and generate predictive models, making them well-suited for identifying novel subgroups based on metabolic and clinical characteristics^9^. Several studies have investigated the application of ML techniques to classify subtypes of obesity and MetS^10–14^.

Currently, individuals can be stratified into four groups based on obesity status and metabolic profiles: metabolically healthy non-obese (MHNO), metabolically healthy obese (MHO), metabolically unhealthy obese (MUO), and metabolically unhealthy non-obese (MUNO)^15^. In our study, we aim to develop an unsupervised machine learning framework to re-clustering of samples within the three groups of MHO, MUO, and MUNO with the goal of gaining deeper insights into their metabolic diversity. This approach offers several advantages: it captures metabolic heterogeneity beyond obesity status, identifies both shared and unique patterns among the groups, provides unbiased and refined subgroup definitions, and enhances risk stratification by focusing on underlying metabolic traits rather than relying solely on anthropometric measures^16^.

Traditional classification methods often fail to capture the heterogeneity within obese populations, highlighting the importance of utilizing machine learning algorithms to uncover hidden patterns and complex relationships within the data^17–20^.

Addressing the complexities of analyzing obesity and metabolic syndrome in children and adolescents, this study proposes a refined classification system based on an unsupervised machine-learning approach. By focusing on key clinical variables that capture the multidimensional relationship between obesity progression and metabolic processes, and utilizing advanced artificial intelligence (AI) techniques, the research aims to identify distinct obesity phenotypes. The objective of this study was to explore whether unsupervised clustering of anthropometric and metabolic profiles can reveal more specific subgroups among children and adolescents. Gaining such insights could serve as an initial step toward developing classification frameworks that may ultimately support improved risk stratification and personalized interventions.

## Method

### Study design

The data for this study were obtained from three nationwide, multicenter, cross-sectional school-based surveillance projects in Iran. These projects, collectively known as the Childhood and Adolescence Surveillance and Prevention of Adult Non-Communicable Disease (CASPIAN) studies included CASPIAN I (2003–2004), CASPIAN III (2009–2010), and CASPIAN V (2015–2016). Each study was conducted across more than 20 provinces and involved students and one of their parents^21–23^. This study was approved by the Ethics Committee of Isfahan University of Medical Sciences (Research ethics code: IR.ARI.MUI.REC.1402.286).

Informed oral assent and written consent forms were collected from participants and parents, respectively, after a description of the aims and protocol of the study. This study was conducted in accordance with the principles outlined in the Declaration of Helsinki, and all procedures involving human participants were approved by the Ethics Committee of Isfahan University of Medical Sciences, Isfahan.

After excluding missing data, the sample sizes for CASPIAN I, III, and V were 4811, 5625, and 14,286, respectively. The proportion of females in these datasets was 52.6%, 49.0%, and 47.3%, respectively. Table 1 presents the distribution of samples classified as MHO, MUNO, and MUO within each dataset.

**Table 1:** Characteristics of dataset.

Dataset	Original data	Missing removal	Constraint age 7–18	MHO	MUNO	MUO
CASPIAN I	4811	4670	4465	656	114	206
CASPIAN III	5625	2801	2801	313	157	185
CASPIAN V	14,286	3732	3732	593	240	155

Metabolic healthy-obese (MHO), metabolic unhealthy-non obese (MUNO), metabolic unhealthy-obese (MUO).

A team of qualified healthcare providers conducted the physical examinations following established protocols and utilizing calibrated instruments. The study’s overall protocols were primarily based on the World Health Organization’s Global School-based Student Health Survey, with detailed descriptions available in earlier publications^21–23^.

To address the status of obesity and MetS (MHO, MUO, MUNO), we considered three anthropometric indicators (height, weight, and waist circumference) and seven clinical parameters: cholesterol, fasting blood sugar (FBS), triglycerides (TG), high-density lipoprotein (HDL), low-density lipoprotein (LDL), systolic blood pressure, and diastolic blood pressure. Sex and age were also included as variable.

During the preprocessing stage, missing values were handled within each dataset before merging the CASPIAN I, III, and V datasets. The combined data were then categorized into three developmental age groups: 7–10, 11–14, and 15–18 years. These groups align with commonly used developmental stages in pediatric and adolescent research, roughly corresponding to middle childhood, early adolescence, and late adolescence^24–26^. Outliers were removed in two steps: first, age-specific outliers were identified based on the lowest and highest percentiles for each variable; second, a multivariate outlier detection technique, Isolation Forest, was applied with a contamination rate of 0.05.

To enhance the identification of participants with obesity and/or MetS, we applied well-established diagnostic criteria. Body Mass Index (BMI) was assessed according to WHO growth standards, classifying individuals as underweight (below the 5th percentile), overweight (85th–95th percentile), and obese (above the 95th percentile), with adjustments made for age and sex. The definition of metabolic syndrome (MetS) in children and adolescents includes several key components: (I) Abdominal Obesity: Often measured by waist circumference (WC), with cut-off values varying by age, sex, and ethnicity. (II) Dyslipidemia: Characterized by elevated triglycerides (TG) and low high-density lipoprotein cholesterol (HDL-C). (III) Hypertension: Defined as elevated blood pressure (BP) based on age- and sex-specific percentiles. (IV) Disturbed Glucose Metabolism: Includes impaired fasting glucose or insulin resistance^27,28^.

In the current study, subjects were classified as having MetS if they met at least three of the following criteria, based on the Adult Treatment Panel III (ATP III) criteria modified for the pediatric age group^29^: (A) abdominal obesity defined as a waist-to-height ratio equal to or more than 0.5^30^, (B) TG > = 100 mg/dL, (C) HDL-C < 40 mg/dL (for boys age 15–18 the cut-off was < 45 mg/dL), (D) Elevated FBG > = 100^31^, (E) Elevated blood pressure defined as either high systolic blood pressure (SBP) ( > = 90th percentile for age, sex and height) or high diastolic blood pressure (DBP) (≥ 90th percentile for age, sex and height)^32^.

From the entire dataset, individuals identified as overweight and/or having MetS (MHO, MUO, MUNO), based on the aforementioned criteria, were selected as the input for this study. While there is a slight difference between the definitions of overweight and obesity, the term “obesity” is used throughout this study to refer to both conditions. The distribution of variables with regard to age group categories and the metabolic-obesity status of individuals is presented in Table 2. We analyzed factors such as family history of hypertension, diabetes, obesity, osteoporosis, stroke, and malignancy, as well as smoking status, physical activity (classified as more or less than 2 h per day), and screen time to identify differences among the subgroups.

**Table 2:** Distribution of variables with regard to the age group categories and the metabolic-obesity status of individual.

	Age Group1 (MHNO)	Age Group1 (MUNO, MUO, MUNO)	Age Group2 (MHNO)	Age Group2 (MUNO, MUO, MUNO)	Age Group3 (MHNO)	Age Group3 (MUNO, MUO, MUNO)
Number	1763	382	2990	787	2490	594
Male/Female (Girl%)	807/956 (54.2%)	211/171 (55.2%)	1467/1523 (51%)	392/395 (49.8%)	1272/1218 (49.0%)	308/286 (51.9%)
Weight (kg)	25.8 ± 4.5	34.5 ± 6.7	38.9 ± 8.6	52.4 ± 9.7	52.9 ± 8.9	66.9 ± 11.3
Height (cm)	130.4 ± 8.2	132.6 ± 8.5	150.0 ± 10.4	151.7 ± 8.5	164.3 ± 9.4	163.3 ± 8.7
Waist (cm)	56.4 ± 4.7	64.7 ± 7.4	63.6 ± 6.7	74.4 ± 8.2	69.6 ± 6.8	80.6 ± 9.2
Systolic Blood Pressure (mmHg)	92.2 ± 11.0	97.2 ± 11.2	98.5 ± 10.9	103.2 ± 11.0	104.8 ± 11.6	109.4 ± 11.2
Diastolic Blood Pressure (mmHg)	58.1 ± 9.3	61.6 ± 9.0	62.6 ± 9.2	65.3 ± 9.9	66.6 ± 9.5	70.5 ± 9.6
Fasting blood sugar (mg/dl)	84.7 ± 10.1	88.0 ± 10.4	85.9 ± 9.8	88.0 ± 10.6	84.5 ± 10.3	88.4 ± 11.5
Triglyceride (mg/dl)	82.7 ± 30.4	96.8 ± 38.1	85.4 ± 32.7	97.5 ± 37.3	83.4 ± 30.7	100.0 ± 34.3
Cholesterol (mg/dl)	150.6 ± 27.6	157.4 ± 26.6	150.7 ± 25.2	153.6 ± 24.7	143.5 ± 26.7	147.2 ± 25.6
LDL (mg/dl)	88.5 ± 28.2	96.6 ± 28.4	88.3 ± 25.8	93.5 ± 26.4	81.6 ± 25.0	88.5 ± 25.5
HDL (mg/dl)	45.6 ± 10.4	44.7 ± 10.6	45.7 ± 10.7	43.5 ± 10.1	45.1 ± 10.1	41.5 ± 9.1

Metabolic healthy-non obese (MHNO), metabolic healthy-obese (MHO), metabolic unhealthy-non obese (MUNO), metabolic unhealthy-obese (MUO).

### Clustering step

Machine learning has emerged as a powerful tool in data analysis, particularly in the realm of clustering, which involves grouping similar data points based on inherent patterns and characteristics. The Gaussian Mixture Model (GMM) is a probabilistic clustering method that models data as a mix of Gaussian distributions, allowing flexible, interpretable clustering of complex datasets with varied shapes and sizes^33^.

To improve the effectiveness of clustering algorithms like GMM, dimensionality reduction techniques such as Principal Component Analysis (PCA) are often employed. PCA transforms high-dimensional data into a lower-dimensional space while retaining as much variance as possible, thereby simplifying the dataset and facilitating visualization and analysis. This reduction is especially critical in high-dimensional contexts where the “curse of dimensionality” can hinder clustering performance. By applying PCA prior to clustering, researchers can enhance the quality of cluster identification and interpretation^33^.

The combination of PCA with GMM not only aids in revealing latent structures within complex datasets but also supports more accurate classification and prediction tasks across various fields, including healthcare and marketing^33^.

For each age group, we performed clustering by selecting the top five principal components (PCs), which accounted for approximately 80% of the variance in the data. The GMM was then applied to the sample projections on these selected PCs. To determine the optimal number of clusters, we utilized the Davies-Bouldin (DB) index^34^, selecting the cluster configuration that yielded the lowest DB value, indicating the best clustering performance.

### Validation

To validate the clustering results, a two-step evaluation process was conducted. First, a stability analysis was performed using stratified k-fold splitting with 10 to 50 splits to ensure the robustness and consistency of the identified clusters across different data subsets. Stratification was applied to maintain the original distribution of cluster labels within each split, preserving the structure of the data. This method assesses whether the clustering patterns remain stable when a portion of the data is omitted and repeatedly partitioned, ensuring that the results align with those obtained from the complete dataset. Because cluster labels are arbitrary and may differ across repetitions, direct comparison of cluster assignments is not straightforward. To quantify stability, we used Hungarian clustering accuracy, which first aligns clusters from different repetitions using an optimal matching and then calculates the proportion of correctly matched labels. This approach provides a robust measure of clustering consistency across the different data subsets generated by the stratified k-fold splitting and the complete dataset^35,36^.

Second, a predictability test was conducted using a Support Vector Machine (SVM) classifier with 10-fold cross-validation. This step evaluated how effectively the clusters could be predicted by a supervised learning model, serving as an additional measure of their distinctiveness and reliability. Together, these analyses offered complementary insights into the validity and reproducibility of the clustering results.

**Fig. 1:**
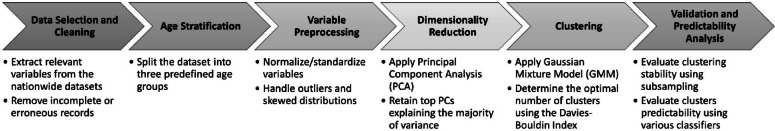
Workflow summarizing the key steps: data cleaning, age stratification, preprocessing, PCA, GMM clustering, and validation.

The main workflow of the study, outlining all key analytical steps from data preprocessing to clustering and validation, is presented in Fig. 1.

### Statistical analysis

Within each age group, we examined the clinical consequences of factors linked to metabolism and obesity across the identified clusters. Categorical variables—including family history of selected diseases, smoking status, physical activity, and screen time—were compared across the identified clusters and the three metabolic-obesity subgroups (MUO, MHO, and MUNO) using the Chi-squared test, with Cramer’s V to estimate effect size. Pairwise comparisons were adjusted using Bonferroni correction to control for multiple testing, and only significant differences (adjusted *p* < 0.05) were reported for clarity.

## Results

**Fig. 2:**
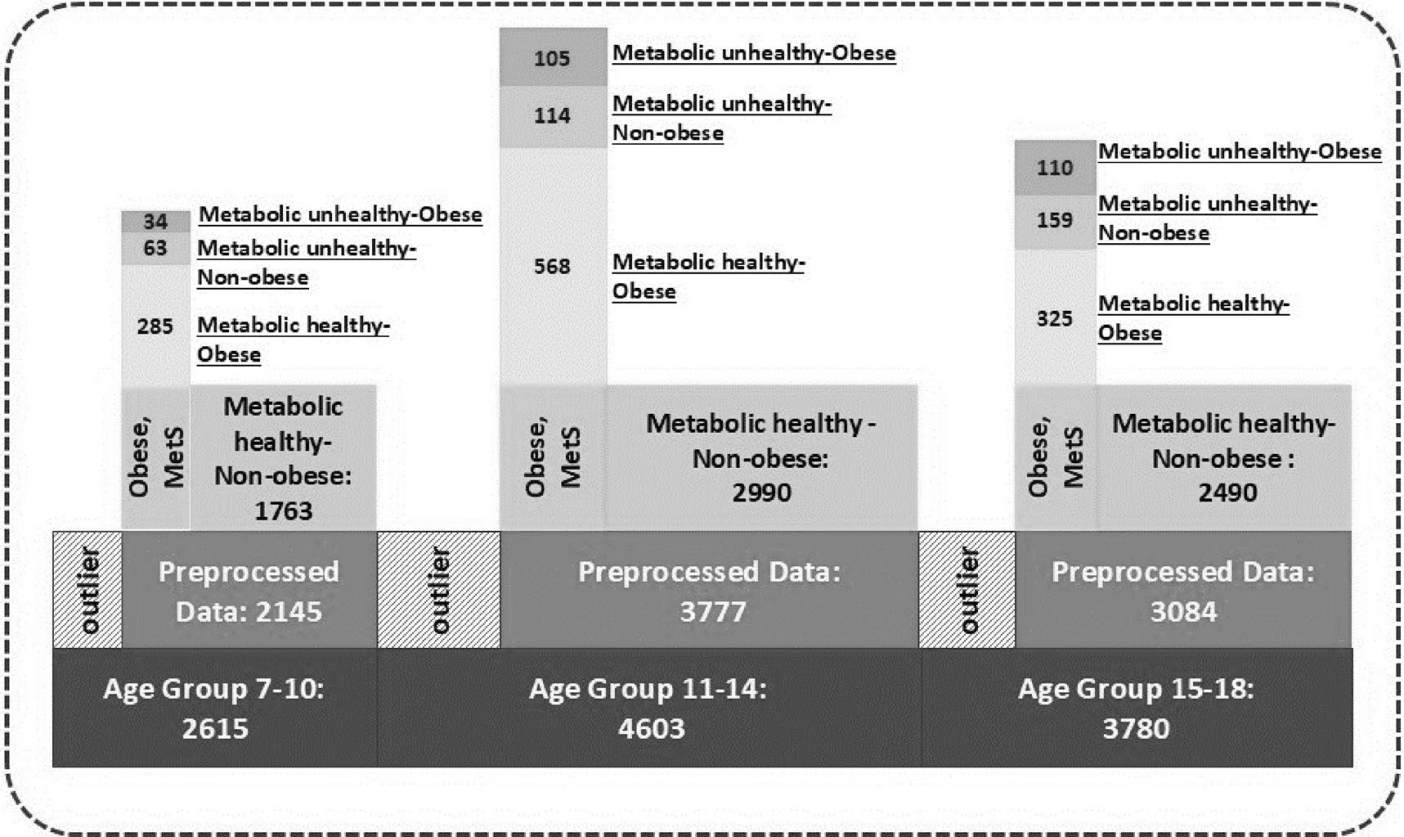
The process of preparing the study population.

After integrating the CASPIAN, I, III, and V datasets and categorizing the data by age group, the initial sample sizes for age groups 1, 2, and 3 were 4,603, 3,780, and 2,615, respectively. Following preprocessing, which involved excluding samples with MHNO status and removing the age variable from the included parameters, the final study comprised 382, 787, and 594 individuals in each age group. The analysis was conducted using 11 anthropometric, biochemical, and physiological variables. A detailed overview of the study population selection process and the statistics of the included variables is provided in Fig. 2; Table 2.

Using the GMM algorithm with five principal components (PCs) and various configurations, the optimal number of clusters for each age group was determined based on Davies-Bouldin (DB index. The results indicated 6, 7, and 6 clusters for age groups 1, 2, and 3, respectively. The cumulative variance plots are provided in Supplementary Fig. 1. Supplementary Fig. 2 presents the DB index plot used to select the optimal number of clusters.

**Fig. 3:**
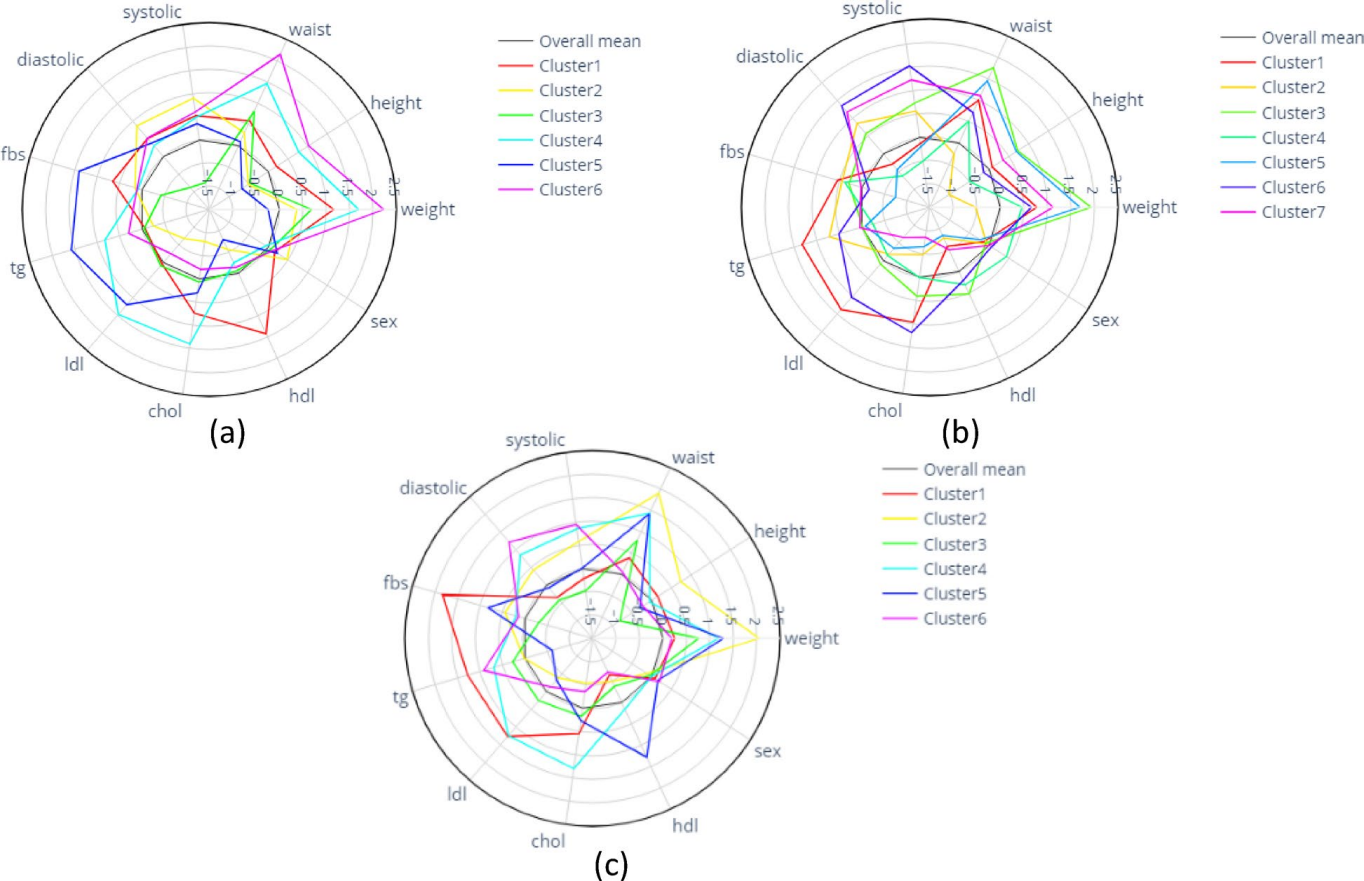
Cluster profile of (**a**) age group 1, (**b**) age group 2, and (**c**) age group 3.

Radar plots of clusters in each age group are shown in Fig. 3, with more detailed radar plots and bar plots represented in Supplementary Figs. 3–8. Each cluster is characterized by specific symptom profiles, which are briefly described below.

### Characteristics of age group 1 (7–10 years)

In Age Group 1 (7–10 years), six distinct clusters were identified based on key metabolic and anthropometric variables:

- Cluster 1 (High Weight and Lipid Cluster): Characterized by elevated weight, cholesterol, and HDL, indicating lipid-related metabolic risks.
- Cluster 2 (Hypertensive Cluster): Defined by high systolic and diastolic blood pressure (BP), reflecting a hypertension-prone group.
- Cluster 3 (High Weight and Waist Cluster): Dominated by waist circumference and weight, suggesting central obesity as the primary concern.
- Cluster 4 (High Anthropometric and Lipid Cluster): Exhibits elevated anthropometric measures, TG, LDL, and cholesterol, highlighting a mixed metabolic risk profile.
- Cluster 5 (High Metabolic Syndrome Risk Cluster): Shows overall elevation in multiple MetS risk factors, indicating a high-risk metabolic profile.
- Cluster 6 (Obesity-Dominant Cluster): Marked by high anthropometric measures, with obesity as the primary risk driver.

### Characteristics of age group 2 (11–14 years)

In Age Group 2 (11–14 years), seven distinct clusters were identified, each highlighting unique metabolic and anthropometric characteristic:

- Cluster 1 (High Weight, Waist, and Lipid Cluster): Elevated weight, waist circumference, TG, LDL, and cholesterol, reflecting a lipid-centric metabolic risk profile.
- Cluster 2 (Hypertensive and Glucose Cluster): Defined by high BP, TG, and fasting blood sugar (FBS), suggesting hypertension and glucose metabolism issues.
- Cluster 3 (High Anthropometric and Moderate MetS Risk Cluster): Elevated anthropometric measures with moderate MetS risk factors, indicating a balanced yet concerning profile.
- Cluster 4 (Central Obesity Cluster): Dominated by high waist circumference, emphasizing central obesity.
- Cluster 5 (High Anthropometric, Low MetS Risk Cluster): Elevated anthropometric measures but lower MetS risk factors, indicating physical risk markers with minimal biochemical disturbances.
- Cluster 6 (High BP and Lipid Cluster): Combined high BP, TG, LDL, and HDL, representing a mixed hypertension and lipid risk profile.
- Cluster 7 (High Anthropometric and BP Cluster): Elevated anthropometric measures alongside high BP, suggesting a combined physical and vascular risk pattern.

### Characteristics of age group 3 (15–18 years)

In Age Group 3, the clustering patterns highlight a more pronounced interplay between anthropometric measures and metabolic risk factors, reflecting physiological stabilization typical of late adolescence:

- Cluster 1 (Metabolic Risk Cluster): Elevated LDL, TG, and FBS, indicating significant metabolic dysregulation.
- Cluster 2 (Central Obesity Cluster): Defined by high waist circumference, emphasizing abdominal obesity.
- Cluster 3 (Anthropometric-Moderate Metabolic Cluster): High anthropometric measures with moderated blood pressure (BP) and fasting blood sugar (FBS), indicating a distinct profile of physical and metabolic characteristics.
- Cluster 4 (Severe Metabolic Syndrome Cluster): Elevated weight, waist, TG, LDL, cholesterol, and BP, representing severe metabolic and anthropometric risks.
- Cluster 5 (Hypertensive Cluster): Elevated BP and TG, highlighting hypertension as the dominant risk factor.
- Cluster 6 (Obesity-Glucose Cluster): Elevated weight, waist, FBS, and HDL, reflecting obesity-driven glucose dysregulation and lipid imbalance.

### Verification of clusters

Figure 3 illustrates a comparison between the identified clusters and the predefined labels of MHO, MUO, and MUNO.

Further validation of the clusters across three age groups yielded mean clustering accuracies of 76.3%, 65.5%, and 52% in the stability test, respectively. Additionally, the predictability test demonstrated an average accuracy, macro-averaged precision, recall, and F1 exceeding 87%, 89%, 90%, and 88%, respectively across all three age groups, highlighting the robustness and reliability of the clustering results. Evaluation using other classification methods yielded results in a similar range. Further details of these experiments are provided in Supplementary Table 1.

Further analysis and comparisons of variables, such as family disease history, physical activity, and screen time, among the identified clusters and obesity-MetS categories (MHO, MUO, MUNO) are provided in Supplementary Tables 2–4.

Supplementary Fig. 9 illustrates the sex distribution across the identified clusters within three age groups. Supplementary Fig. 10 provides an additional assessment to verify the balanced distribution of the dataset sourced from CASPIAN I, III, and V across different clusters. Supplementary Fig. 11 presents T-SNE plots depicting two-dimensional representations of the clusters across the three age groups.

## Discussion

In this study we successfully identified diverse and complex subgroups of children and adolescents with obesity and metabolic syndrome. By applying GMM clustering in combination with PCA for dimensionality reduction, we precisely characterized the metabolic and anthropometric heterogeneity across three age groups (7–10, 11–14, and 15–18 years).

Cluster analysis revealed 6, 7, and 6 distinct subgroups in the 7–10, 11–14, and 15–18 age groups, respectively. In the youngest group (7–10 years), clusters were primarily characterized by anthropometric variables such as weight and waist circumference.

In the 11–14 age group, increased cluster heterogeneity coincided with pubertal changes, and blood pressure emerged as a key differentiator. This finding is clinically important because it coincides with early adolescence, a developmental stage marked by hormonal and behavioral changes that can amplify cardiometabolic risk^37–40^. By late adolescence (15–18 years), metabolic parameters such as fasting blood glucose (FBS), triglycerides (TG), LDL cholesterol, and blood pressure became dominant in shaping cluster structure, indicating physiological stabilization and greater cardiometabolic risk.

Our clustering revealed some non-intuitive profiles. For instance, in the 15–18 years group, we identified an “obesity–glucose” cluster where adolescents exhibited high anthropometric indices coupled with elevated fasting blood sugar but relatively preserved lipid profiles. This suggests that dysglycemia may emerge independently of dyslipidemia in late adolescence. Similarly, in younger children (7–10 years), a “hypertensive cluster” was identified, underscoring that elevated blood pressure can be an early isolated risk factor even before full metabolic syndrome develops.

These results suggest that relying on conventional obesity or MetS categories may overlook high-risk subgroups. From a clinical perspective, targeted screening of adolescents in the 11–14 years age group may be especially important, as this developmental stage appears most heterogeneous. From a public health perspective, early detection of clusters such as “isolated hypertension” or “obesity–glucose dysregulation” can guide age-specific prevention strategies and personalized interventions, ultimately helping to curb the progression of pediatric metabolic disorders into adulthood.

We also observed that clusters primarily defined by anthropometric measures, such as weight and waist circumference, were consistently present across all age groups. However, the combination of these measures with other metabolic parameters varied within each cluster. For example, in younger children (7–10 years), some clusters were mainly characterized by elevated weight and lipid profiles, while older age groups showed more complex interactions involving blood pressure, glucose, and lipid disturbances.

Our findings are consistent with international research underscoring the metabolic heterogeneity within obese populations. For instance, a multicenter study by Ziwei et al. (2021) involving 2,495 Chinese adults applied unsupervised machine learning algorithms to clinical variables and identified four metabolically distinct clusters (MHO, HMO-U, HMO-I, and LMO) thereby highlighting the complexity of metabolic disorders and reinforcing the need for more accurate diagnostic and therapeutic strategies^10^.

In 2022, a retrospective study of 2,567 obese individuals aged ≤ 18 years in Italy employed machine learning models to identify clinical and biochemical predictors of metabolic health status, revealing that the IGF-1 z-score standard deviation (zSDS) was a significant marker for distinguishing metabolically healthy obesity (MHO) from metabolically unhealthy obesity (MUO), thereby underscoring its utility in clinical risk assessment and personalized obesity management^27^. Collectively, such studies demonstrate the critical role of advanced analytical approaches in elucidating the heterogeneity of metabolic disorders and guiding targeted interventions.

Previous studies have utilized various machine learning algorithms and clinical variables to identify metabolic subgroups among individuals with obesity or MetS; for example, a multicenter study applied k-means and two-step clustering techniques using three clinical indicators—the area under the curve (AUC) for glucose and insulin during an oral glucose tolerance test (OGTT), and serum uric acid levels—to define distinct metabolic phenotypes^10^. In contrast, our study employed the Gaussian Mixture Model (GMM), a probabilistic clustering technique that models data as a combination of multiple Gaussian distributions, offering greater flexibility in identifying clusters of varying shapes and sizes and providing probabilistic assignments that enhance interpretability and reveal nuanced data structures, especially where traditional methods like k-means may be insufficient^33^.

A key strength of this study lies in its novel application of unsupervised machine learning to identify previously unrecognized subgroups of obesity and metabolic syndrome among Iranian children and adolescents, using a comprehensive set of diagnostic components—including fasting blood glucose, triglycerides, HDL cholesterol, waist circumference, and both systolic and diastolic blood pressure—unlike prior studies that relied on more limited indicators^12^.

This study should be interpreted as an exploratory, cross-sectional analysis aimed at uncovering latent structure rather than establishing definitive phenotypic trajectories. Because the data are cross-sectional, we cannot infer developmental progressions or causal relationships. To minimize the strong confounding effect of age, we stratified participants into three developmental stages, but we acknowledge that this approach fragments the sample and limits comparability across age groups. In addition, although we performed internal validation through stability analysis and SVM-based predictability, the lack of validation in an independent contemporary cohort limits the generalizability of our findings. Future longitudinal studies in large, independent cohorts will be essential to confirm and extend these results.

## Conclusion

This study applied machine learning–based clustering to a large national dataset and highlighted the metabolic and anthropometric diversity among children and adolescents with obesity and/or metabolic syndrome. The identified clusters suggest potential heterogeneity in risk profiles throughout developmental stages, notably during adolescence. While these findings provide preliminary insights beyond traditional obesity and MetS classifications, they should be interpreted with caution due to the cross-sectional design, age-stratified approach, the use of a nationwide dataset, and limited external validation. The findings are meant as a first step toward understanding underlying subgroup structure rather than as a basis for immediate clinical application. Future longitudinal studies and external validation in independent cohorts are required to confirm the stability and clinical relevance of these clusters before they can inform risk stratification or personalized interventions.

## Supplementary Information

Below is the link to the electronic supplementary material.

Supplementary Material 1.

Supplementary Material 2.

## Data Availability

The data that support the findings of this study are available from the corresponding author upon reasonable request.
