# Supplementary material for: Uncovering age-specific subtypes of pediatric obesity and metabolic syndrome using machine learning algorithms
Source: Sci Rep. 2025 Nov 19;15:40722. doi: 10.1038/s41598-025-24524-4 (PMC12630947; doi:10.1038/s41598-025-24524-4)
Supplement: Supplementary file 1 — Supplementary Material 1. [file 41598_2025_24524_MOESM1_ESM.docx]

**SUPPLEMENTARY FIGURES**

Supplementary Figure 1. Cumulative variance explained by principal components, showing that the first five components account for approximately 80% of the total variance. Scree plots of eigen values show almost first five eigen values are above 1.


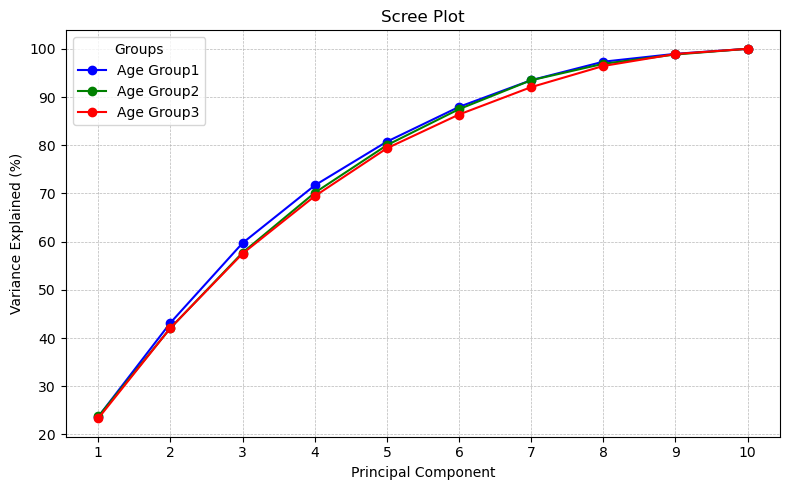

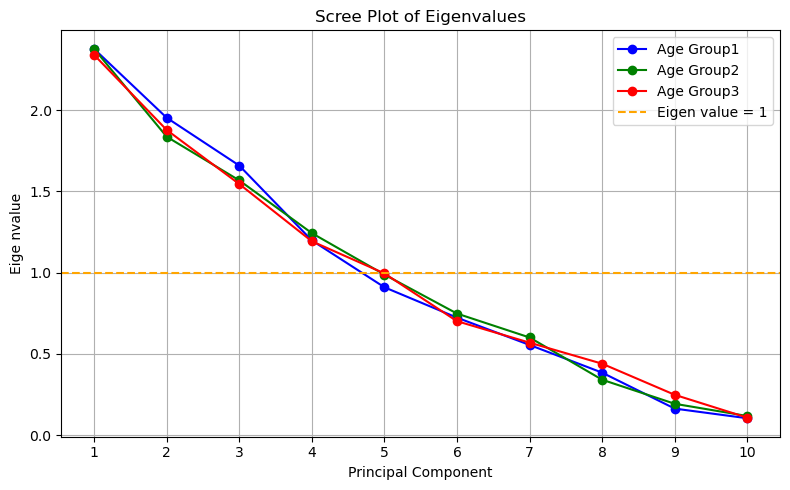

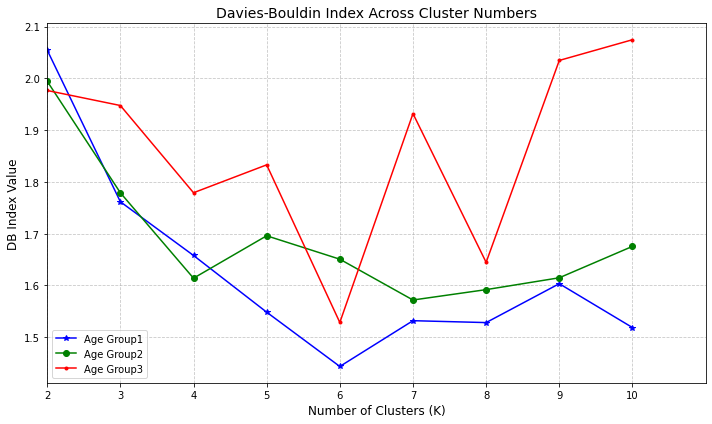


Supplementary Figure 2. Davis-Bouldin index for defining the optimum number of clusters in different age groups.

Cluster 1

Cluster 2

Cluster 3


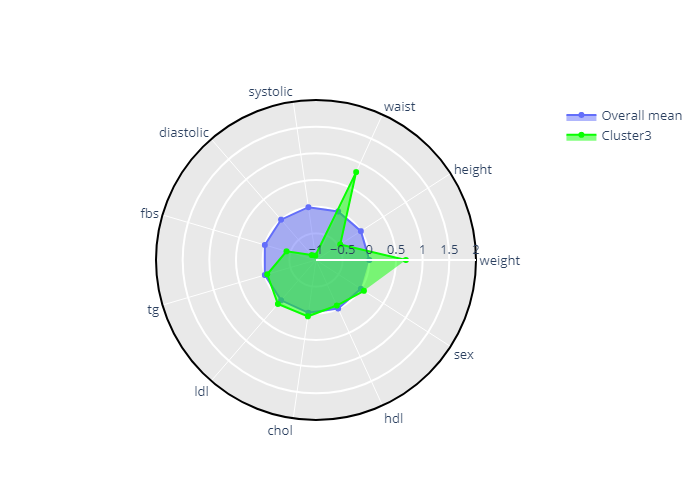

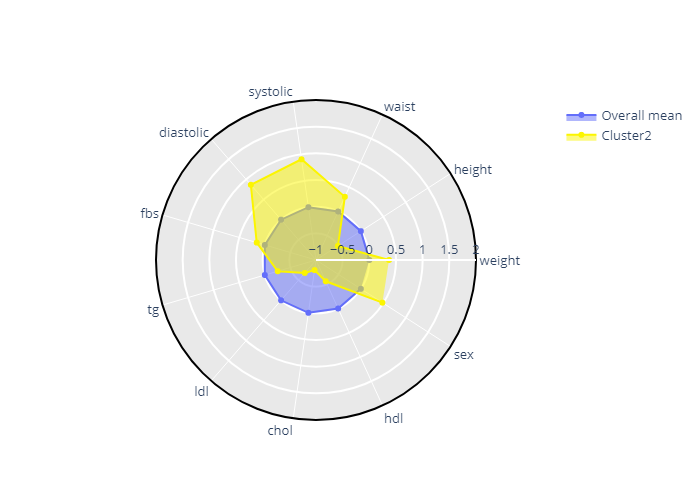

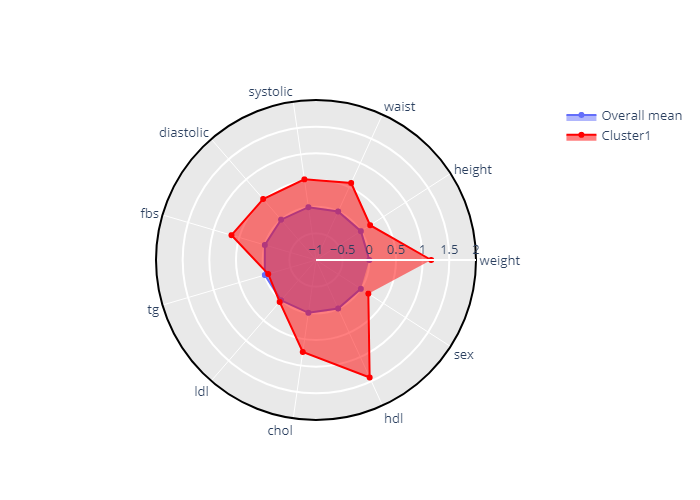

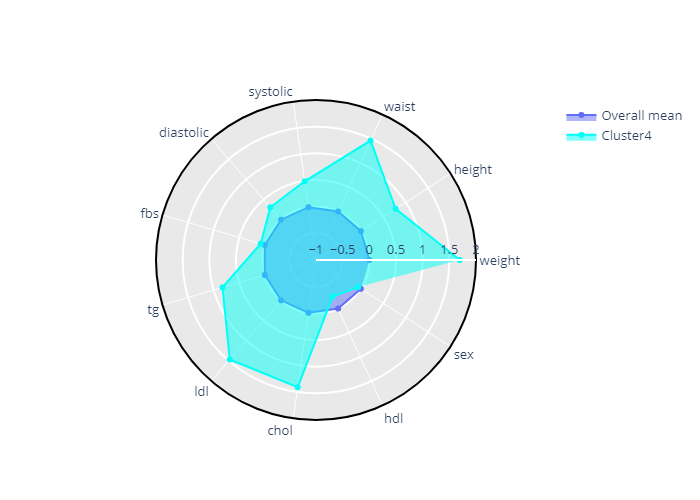

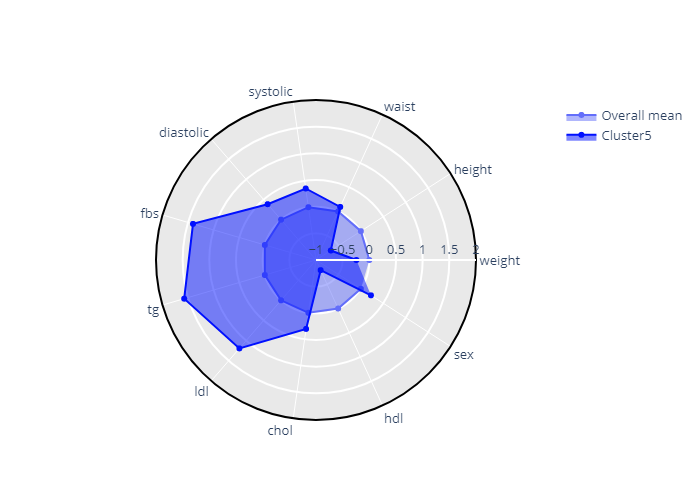

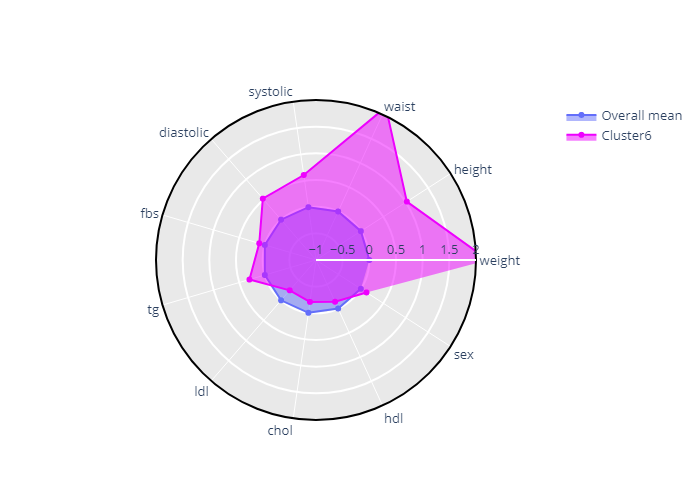


Cluster 4

Cluster 5

Cluster 6

Supplementary Figure 3. Radar plot of clusters in age group 1.


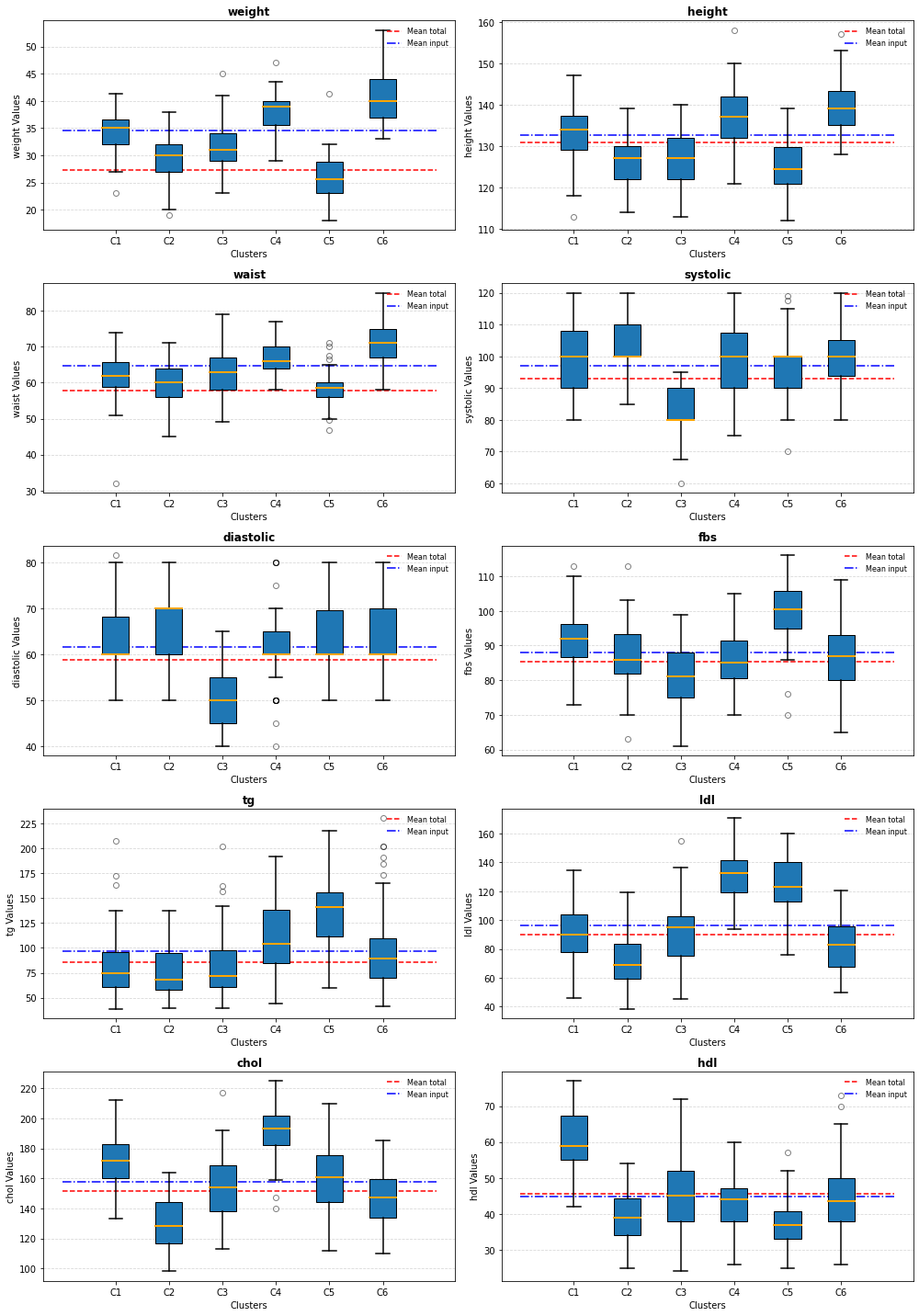


Supplementary Figure 4. Bar plot of clusters in age group 1.


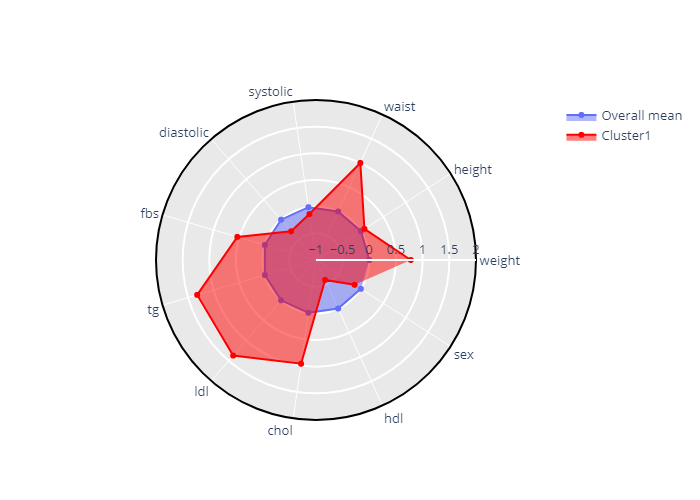

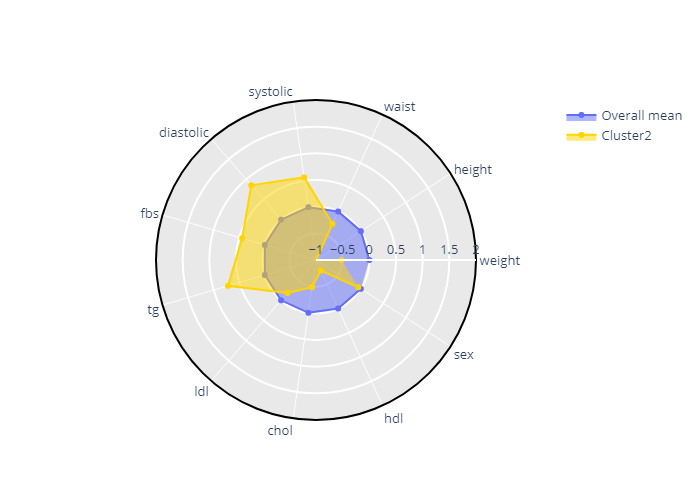

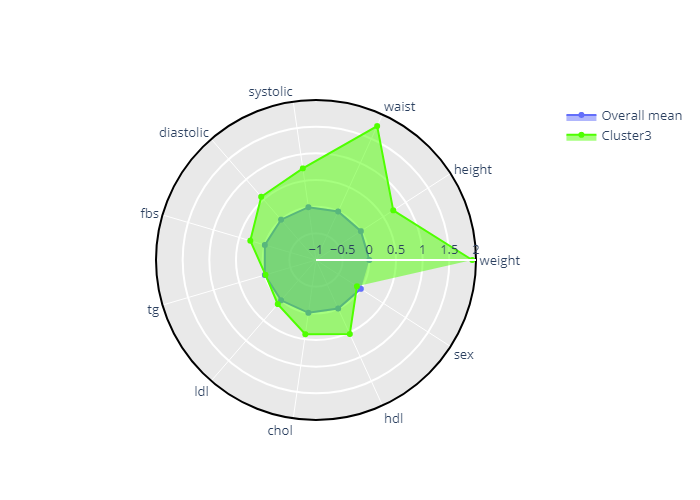

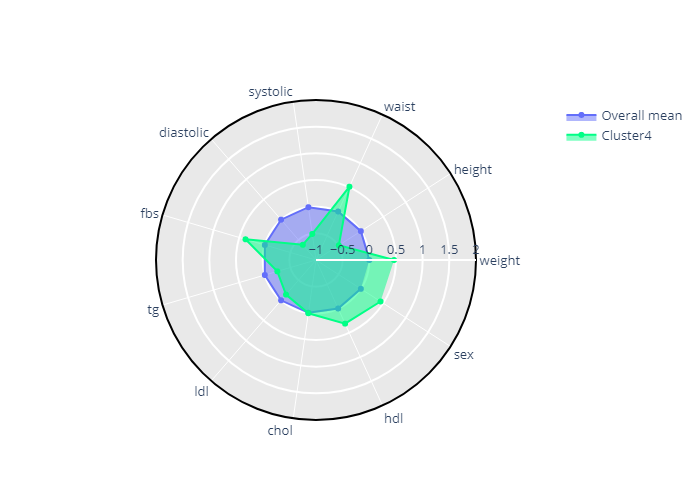

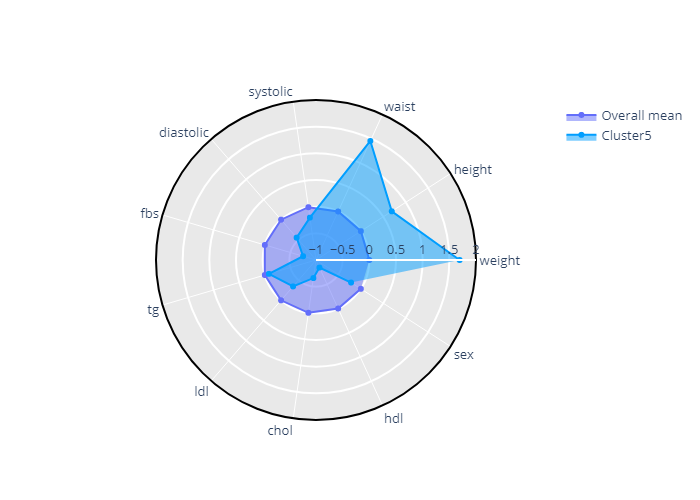

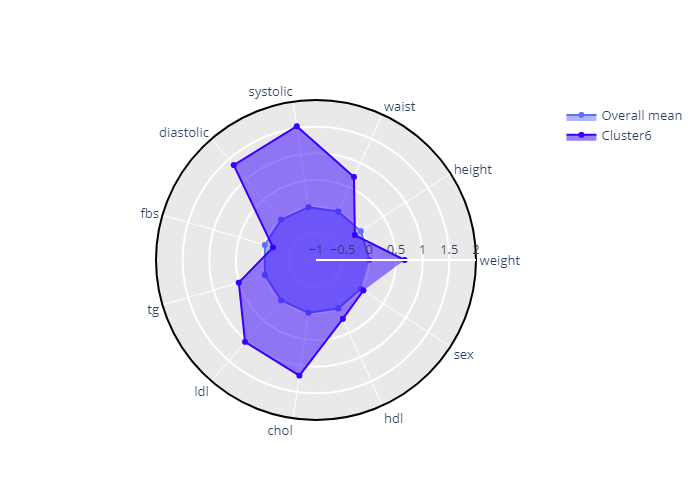

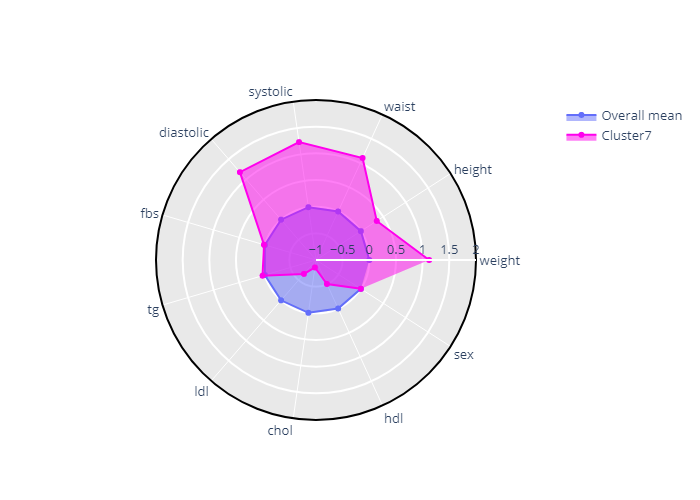


Cluster 1

Cluster 2

Cluster 3

Cluster 4

Cluster 5

Cluster 6

Cluster 7

Supplementary Figure 5. Radar plot of clusters in age group 2.


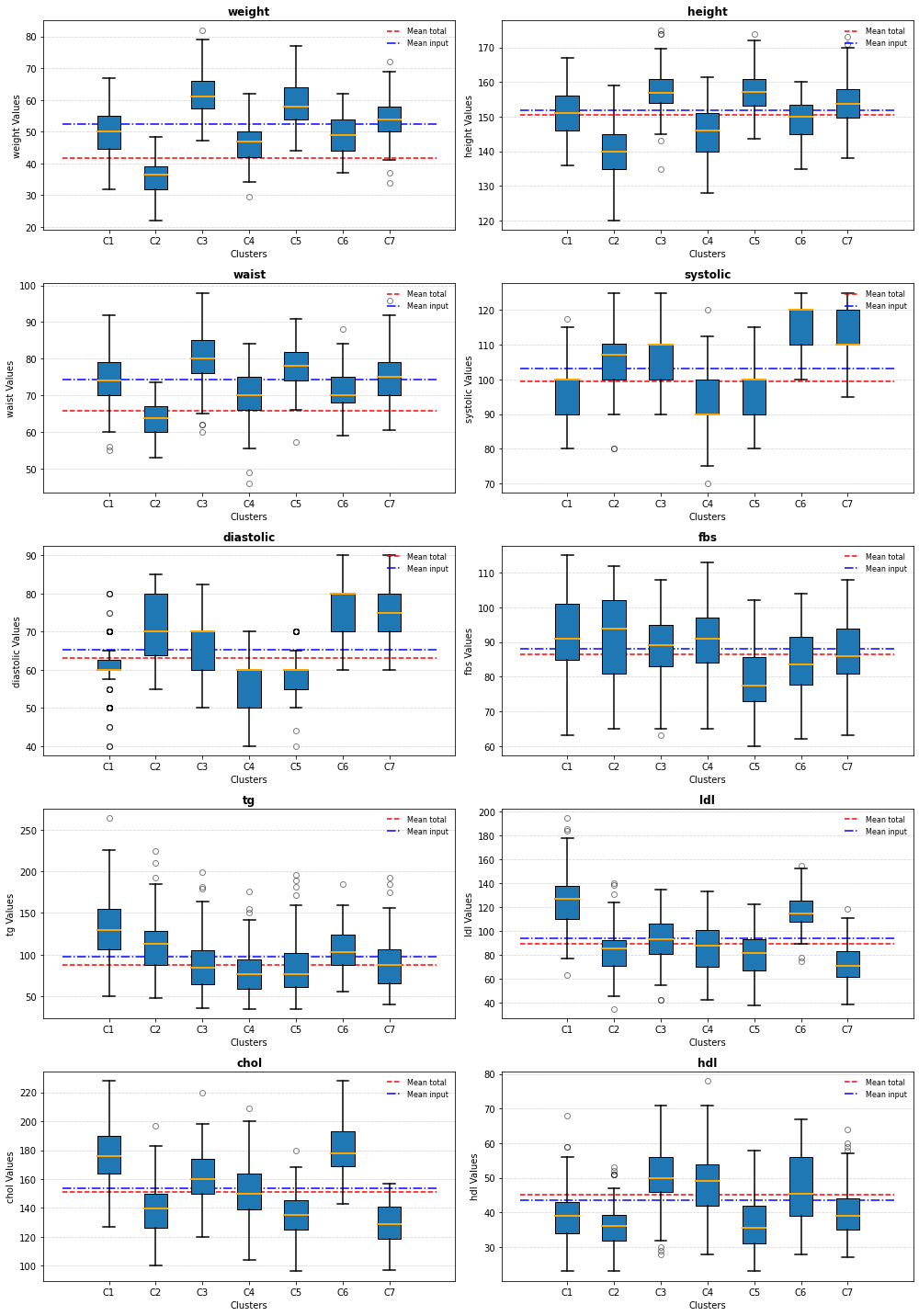


Supplementary Figure 6. Bar plot of clusters in age group 2.


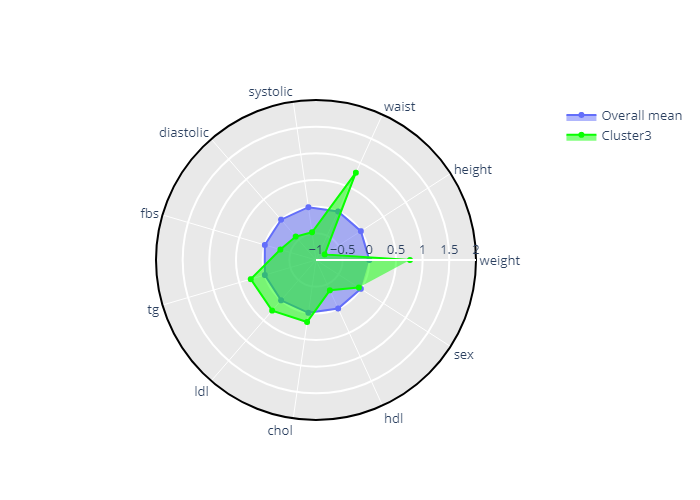

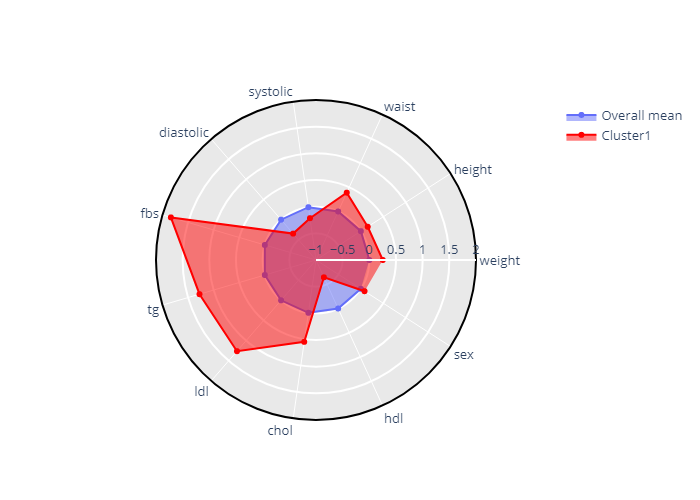

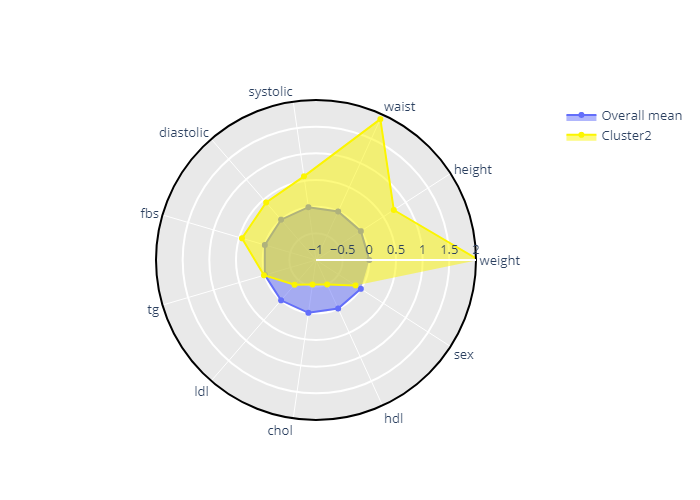

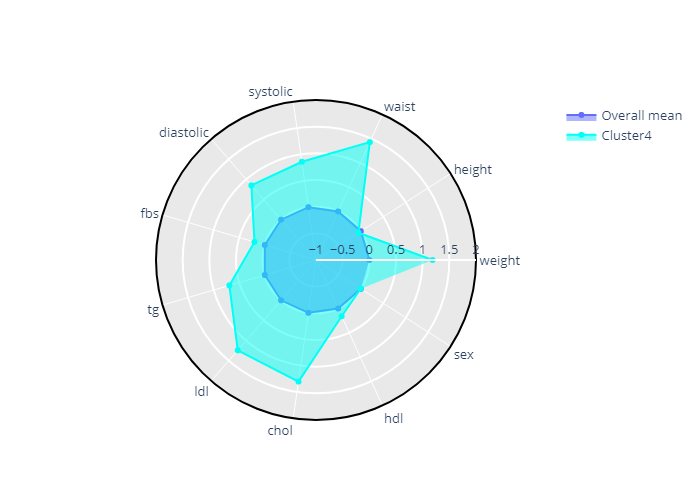

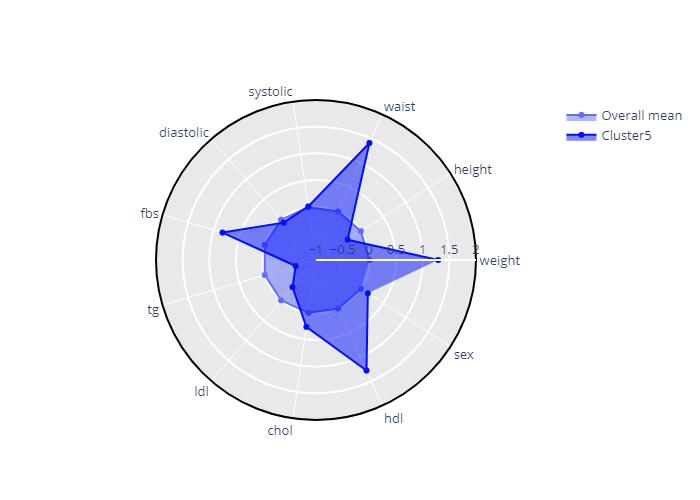

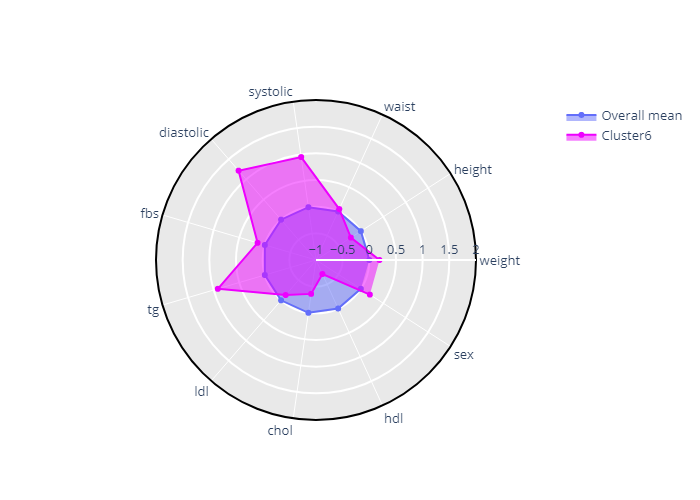


Cluster 1

Cluster 2

Cluster 3

Cluster 4

Cluster 5

Cluster 6

**Supplementary Figure 7.** Profile of clusters in age group 3.


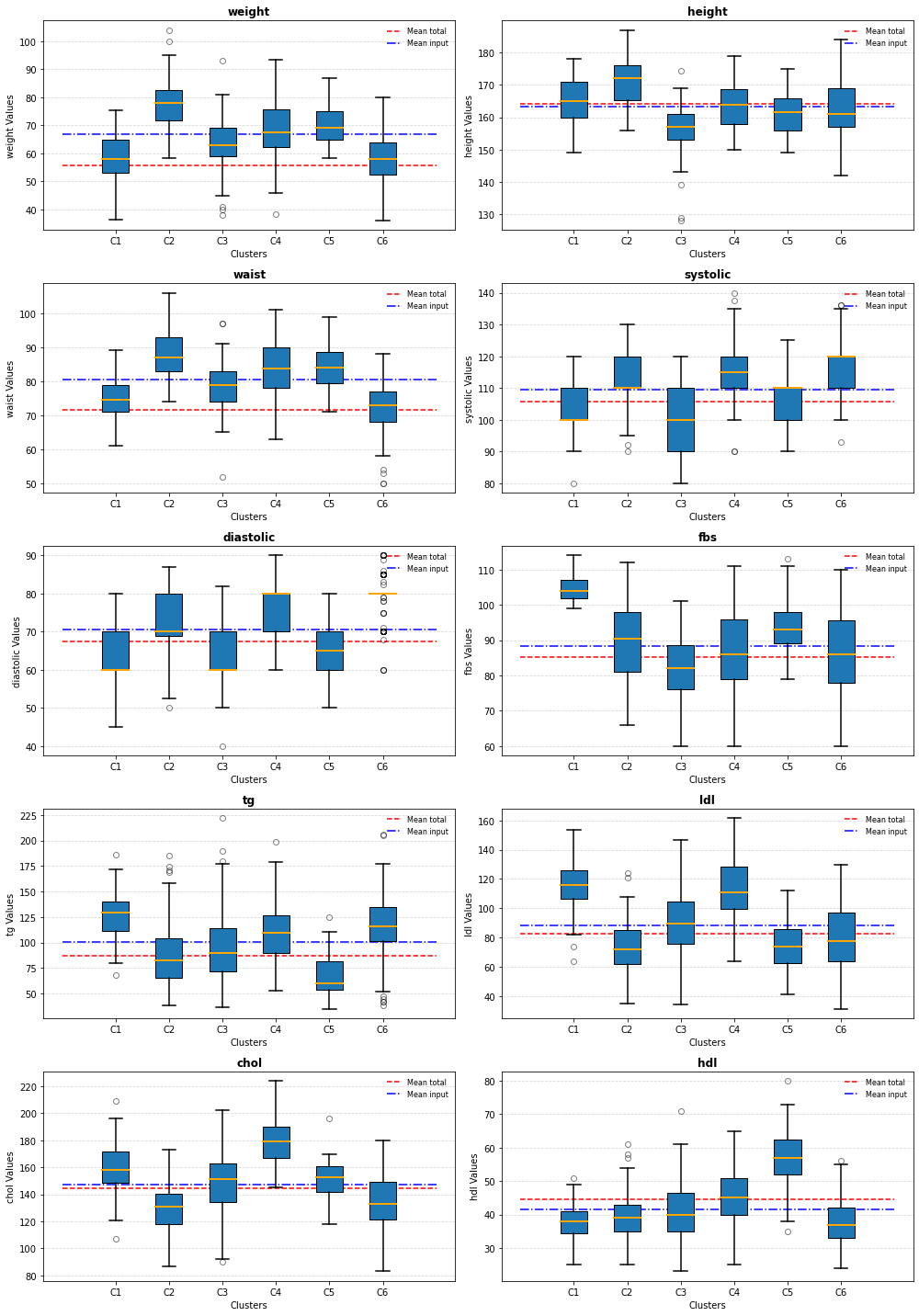


Supplementary Figure 8. Bar plot representing clusters in age group 3. Each plot includes a 'Mean Input' line, showing the average value of the variable across the specific subpopulation (individuals with MUO, MUNO, and MHO). Additionally, a 'Mean Total' line indicates the overall average of the total population in age group 1, which also includes individuals classified as MHNO.


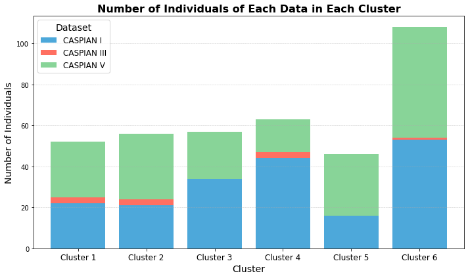

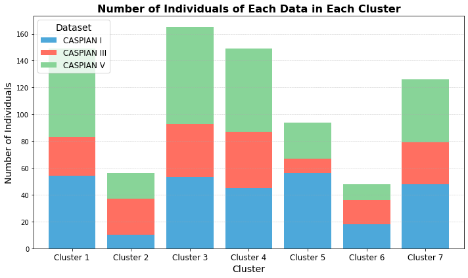

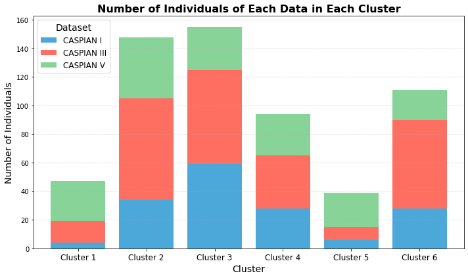


(a)

(b)

(c)

Supplementary Figure 10. Distribution of CASPIAN datasets across clusters in a) age group1, b) age group 2), and c) age group3.


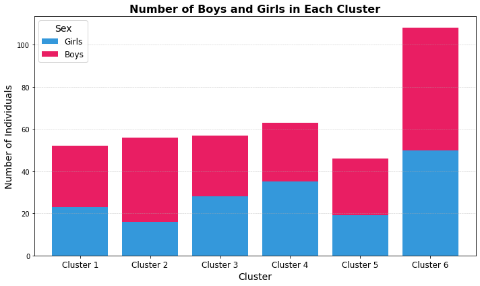

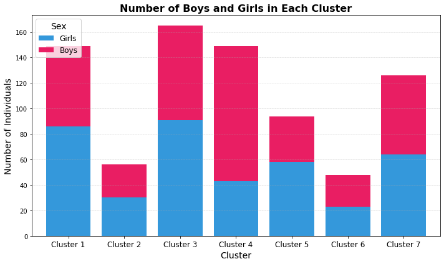

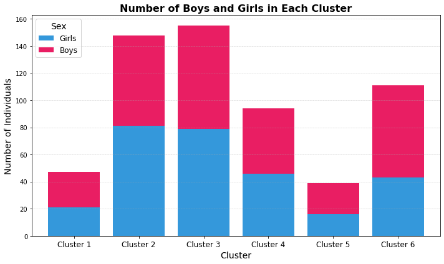


(a)

(b)

(c)

Supplementary Figure 9. Distribution of sex in identified clusters in a) age group 1, b) age group 2, c) age group 3.


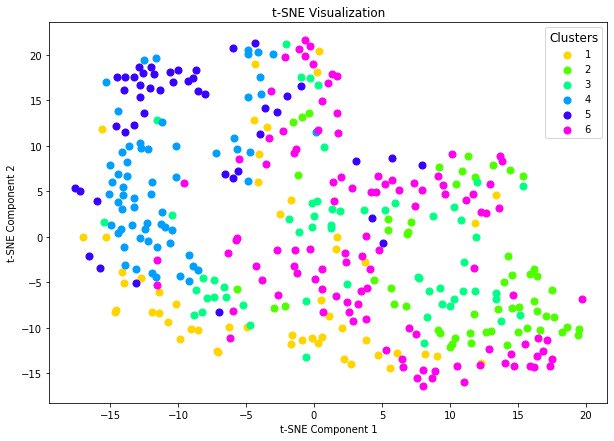

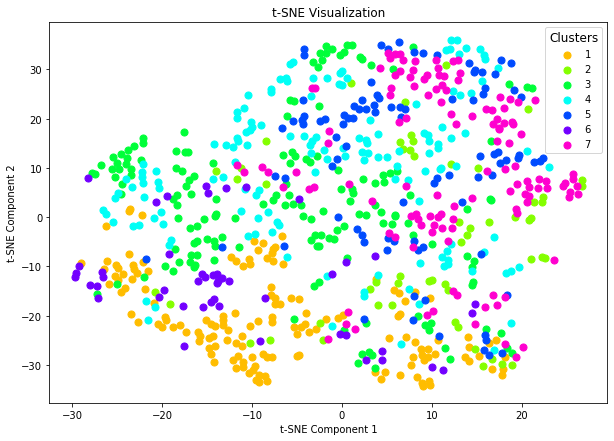

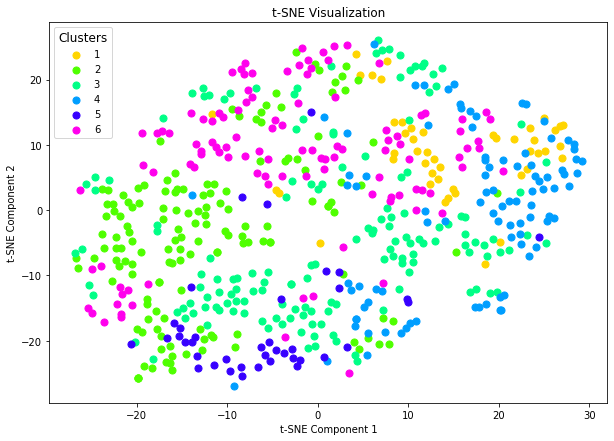


(a)

(b)

(c)

Supplementary Figure 11. T-SNE projection of samples according to defined clusters in a) age group1, b) age group2, and c) age group3.
