# Supplementary material for: Uncovering age-specific subtypes of pediatric obesity and metabolic syndrome using machine learning algorithms
Source: Sci Rep. 2025 Nov 19;15:40722. doi: 10.1038/s41598-025-24524-4 (PMC12630947; doi:10.1038/s41598-025-24524-4)
Supplement: Supplementary file 2 — Supplementary Material 2. [file 41598_2025_24524_MOESM2_ESM.docx]

**SUPPLEMENTARY TABLE**

Supplementary Table 1. Summary of classification performance for cluster predictability using 10-fold cross-validation across three age groups. Metrics include accuracy, macro-averaged precision, recall, and F1-score.

| Age Group1 | | | | |
| --- | --- | --- | --- | --- |
| Model | **Accuracy** | **Precision** | **Recall** | **F1** |
| SVM | 0.895 ± 0.062 | 0.912 ± 0.058 | 0.890 ± 0.071 | 0.893 ± 0.069 |
| Logistic Regression | 0.935 ± 0.024 | 0.945 ± 0.026 | 0.931 ± 0.027 | 0.934 ± 0.026 |
| Random Forest | 0.832 ± 0.056 | 0.839 ± 0.090 | 0.809 ± 0.069 | 0.813 ± 0.077 |
| KNN | 0.790 ± 0.091 | 0.799 ± 0.116 | 0.781 ± 0.111 | 0.779 ± 0.116 |
| Age Group2 | | | | |
| Model | **Accuracy** | **Precision** | **Recall** | **F1** |
| SVM | 0.905 ± 0.033 | 0.920 ± 0.035 | 0.886 ± 0.044 | 0.895 ± 0.041 |
| Logistic Regression | 0.931 ± 0.022 | 0.939 ± 0.021 | 0.917 ± 0.035 | 0.922 ± 0.029 |
| Random Forest | 0.834 ± 0.033 | 0.844 ± 0.049 | 0.807 ± 0.055 | 0.814 ± 0.053 |
| KNN | 0.797 ± 0.043 | 0.826 ± 0.053 | 0.739 ± 0.046 | 0.756 ± 0.048 |
| Age Group3 | | | | |
| Model | **Accuracy** | **Precision** | **Recall** | **F1** |
| SVM | 0.899 ± 0.039 | 0.906 ± 0.036 | 0.872 ± 0.036 | 0.880 ± 0.035 |
| Logistic Regression | 0.897 ± 0.026 | 0.893 ± 0.035 | 0.874 ± 0.042 | 0.876 ± 0.037 |
| Random Forest | 0.843 ± 0.039 | 0.861 ± 0.065 | 0.817 ± 0.056 | 0.826 ± 0.062 |
| KNN | 0.810 ± 0.034 | 0.823 ± 0.071 | 0.774 ± 0.057 | 0.781 ± 0.063 |

Supplementary Table 2. Statistical analysis of disease family history and other variables across six identified clusters and metabolic-obesity status categories within age group 1. Variables include Hypertension (HTN), Sudden Death (SD), Physical Activity (PA), and Screen Time (ST).

|  |  | MUO, MHO, MUNO | | | Clusters 1-6 | | |
| --- | --- | --- | --- | --- | --- | --- | --- |
| Family History | Variable | P value | Effect Size | Pair-wise | P value | Effect Size | Pair-wise |
|  | HTN | 0.881 | 0.03 | - | 0.261 | 0.13 |  |
|  | DIABETES | 0.511 | 0.06 | - | 0.394 | 0.12 |  |
|  | OBESITY | 0.223 | 0.09 | - | 0.027 | 0.18 |  |
|  | OSETOPOR | 0.18 | 0.09 | - | 0.733 | 0.09 |  |
|  | STROKE | 0.534 | 0.06 | - | 0.551 | 0.1 |  |
|  | SD | 0.459 | 0.06 | - | 0.948 | 0.06 |  |
|  | MALIGNAN | 0.535 | 0.06 | - | 0.321 | 0.12 |  |
|  | PA | 0.26 | 0.08 | - | 0.007 | 0.18 | C3C1 |
|  | ST | 0.354 | 0.07 | - | 0.324 | 0.12 |  |
|  | SMOKING | 0.583 | 0.05 | - | 0.221 | 0.14 |  |
|  | Sex | 0.588 | 0.05 | - | 0.085 | 0.16 |  |

Differences in categorical variables were evaluated using the Chi-square test (effect size: Cramer’s V). Post-hoc pairwise comparisons were adjusted using Bonferroni correction (α = .05); only pairs showing significant differences were reported, while exact p-values for non-significant pairs were omitted to improve clarity.

Supplementary Table 3. Statistical analysis of disease family history and other variables across seven identified clusters and metabolic-obesity status categories within age group 2. Variables include Hypertension (HTN), Sudden Death (SD), Physical Activity (PA), and Screen Time (ST).

|  |  | MUO, MHO, MUNO | | | Clusters 1-7 | | |
| --- | --- | --- | --- | --- | --- | --- | --- |
| Family History | Variable | P value | Effect Size | Pair-wise | P value | Effect Size | Pair-wise |
|  | HTN | 0.454 | 0.04 | - | 0.071 | 0.12 | C1C3 |
|  | DIABETES | 0.236 | 0.06 | - | 0.722 | 0.07 | - |
|  | OBESITY | 0.151 | 0.07 | - | 0.141 | 0.11 | - |
|  | OSETOPOR | 0.676 | 0.03 | - | 0.647 | 0.07 | - |
|  | STROKE | 0.305 | 0.05 | - | 0.686 | 0.07 | - |
|  | SD | 0.299 | 0.06 | - | 0.954 | 0.04 | - |
|  | MALIGNAN | 0.002 | 0.12 | MHO vs MUO | 0.307 | 0.1 | - |
|  | PA | 0.124 | 0.07 | - | 0.002 | 0.14 | C1C5, C4C5, C5C2 |
|  | ST | 0.485 | 0.04 | - | 0.663 | 0.07 | - |
|  | SMOKING | 0.23 | 0.06 | - | 0.267 | 0.1 | - |
|  | Sex | 0.53 | 0.04 | - | 0.0 | 0.22 | C3C4, C7C4, C1C4, C4C5, C4C2 |

Differences in categorical variables were evaluated using the Chi-square test (effect size: Cramer’s V). Post-hoc pairwise comparisons were adjusted using Bonferroni correction (α = .05); only pairs showing significant differences were reported, while exact p-values for non-significant pairs were omitted to improve clarity.

Supplementary Table 4. Statistical analysis of disease family history and other variables across six identified clusters and metabolic-obesity status categories within age group 3. Variables include Hypertension (HTN), Sudden Death (SD), Physical Activity (PA), and Screen Time (ST).

|  |  | MUO, MHO, MUNO | | | Clusters 1-6 | | |
| --- | --- | --- | --- | --- | --- | --- | --- |
| Family History | Variable | P value | Effect Size | Pair-wise | P value | Effect Size | Pair-wise |
|  | HTN | 0.041 | 0.1 | - | 0.041 | 0.14 | C1C3 |
|  | DIABETES | 0.696 | 0.03 | - | 0.401 | 0.09 | - |
|  | OBESITY | 0.68 | 0.04 | - | 0.931 | 0.05 | - |
|  | OSETOPOR | 0.499 | 0.05 | - | 0.538 | 0.08 | - |
|  | STROKE | 0.093 | 0.09 | - | 0.382 | 0.09 | - |
|  | SD | 0.792 | 0.03 | - | 0.779 | 0.06 | - |
|  | MALIGNAN | 0.779 | 0.03 | - | 0.703 | 0.07 | - |
|  | PA | 0.307 | 0.06 | - | 0.378 | 0.1 | - |
|  | ST | 0.069 | 0.09 | - | 0.017 | 0.15 | - |
|  | SMOKING | 0.614 | 0.04 | - | 0.124 | 0.12 | - |
|  | Sex | 0.694 | 0.04 | - | 0.154 | 0.12 | - |

Differences in categorical variables were evaluated using the Chi-square test (effect size: Cramer’s V). Post-hoc pairwise comparisons were adjusted using Bonferroni correction (α = .05); only pairs showing significant differences were reported, while exact p-values for non-significant pairs were omitted to improve clarity.
